# Correspondence

**Published:** 1884-04

**Authors:** 


### CORRESPONDENCE.

TANNIC ACID IN THE TREATMENT OF EXCESSIVE GRANULATIONS. —In a conversation with Dr. W. Lindsay on the treatment of the above condition, he mentioned tannic acid as a most efficient remedy, and spoke so highly of it that I determined to try it. The results were so satisfactory that I considered them worth publishing in your JOURNAL.

I have been using the remedy for more than a year, and am so pleased with it that I have dispensed with all other caustics in these cases. It has one great advantage over others, viz.: it does not produce pain even when applied to the most sensitive parts, and so does away with the necessity of tying the animal.

It is applied in solution by means of a camel's hair brush, and should be made to touch the entire surface of the wound. It soon dries and forms a film over the parts, which should be removed daily and new applications made until healing takes place. No bandages nor coverings.

Following is the prescription:

℞

Acid Tannic.............................. ʒi.

Aqua................................. f. ℥i.

M. External use.

J. LINDSEY, D. V. S., HUNTINGTON, L. I.

*To the Editor of the Journal of Comparative Medicine, etc.:*

I beg to draw attention to the bearing of two paragraphs on Actinomycosis in the January number, which are somewhat misleading, though doubtless unintentionally so.

1st. The editorial on the International Veterinary Congress says: "No reference was made in the Congress to the lately described disease, Actinomycosis, which is, no doubt, in many cases mistaken for the disease under consideration."

Now, while the time available for the consideration of Tuberculosis prevented any discussion on its diagnosis and the diseases that may be confounded with it, there was no such oversight nor neglect in Lydtin's report. As showing analogous lesions he first adduces those contagious affections which advance slowly and insensibly, or by successive accessions, and those that extend along the lymphatics; then continues, "We have to add to all these data, that recently we have come to recognize a new infectious malady, *Actinomycosis*, which provokes in the bones lesions analogous to those found in tuberculosis and glanders, namely, vegetations in the diploï, softening and thickening of the bone, caries, nodules, and nodosities in the lips and tongue, purulent and sanious masses in the heart, etc." Again, "The true criterion of this malady ought to be sought in the irritant which provokes the development of the disorder. If we analyze the nodules or nodosities, the osteo-connective tissue vegetation, the ulcers, or the centres of softening, and there find the vegetable parasite called *Actinomyces bovis* (Harz.), we are certain that we have to deal with neither glanders nor tuberculosis, but with Actinomycosis."

2d. The paragraph from the *Medical Record*, entitled *Actinomycosis discovered in American cattle*, conveys the impression that the disease had not been recognized by American veterinarians until their attention was drawn to it by Dr. Belfield. It is surely the case that American veterinarians, as soon as Bollinger's was noticed in the journals, recognized at once the Actinomycosis in the diseased jaws and soft tissues that are, on the whole, not uncommon. And it can only have been by an oversight that the editor omitted to qualify the paragraph in question, exonerating the profession from the charge of ignorance therein implied. For myself I may state that not only have I taught the true nature of these morbid products in my lectures, but I have secured successful results by the excision of the diseased masses and the treatment of the cavities with iodized phenol. A case having come into my hands opportunely in January, 1883, I carried a number of microscopic slides to the meeting of the Tompkins County Medical Society, February 21, 1883, and upon these demonstrated the nature and treatment of the disease to the members. Dr. Sackett, Secretary of the Society, furnishes me with the following quotation from the minutes of that meeting: "Professor Law favored the Society with a clear statement of the nature and symptoms of Actimonycosis, occurring in various animals and sometimes in man, and exhibited to us the cells of the fungus growth under the microscope."

Respectfully, JAMES LAW,

Professor Veterinary Medicine,

Cornell University, Ithaca, N. Y.

